# A Comparison of Particulate Exposure Levels during Taxi, Bus, and Metro Commuting among Four Chinese Megacities

**DOI:** 10.3390/ijerph19105830

**Published:** 2022-05-10

**Authors:** Ying Zhang, Zhengdong Huang, Jiacheng Huang

**Affiliations:** School of Architecture and Urban Planning, Shenzhen University, 3688 Nanhai Avenue, Shenzhen 518060, China; y.zhang@szu.edu.cn (Y.Z.); 1800325004@email.szu.edu.cn (J.H.)

**Keywords:** particulate matter (PM), commuting exposure, spatiotemporal information, transport microenvironments (TMEs), Chinese megacities

## Abstract

Exposure to inhalable particulate matter pollution is a hazard to human health. Many studies have examined the in-transit particulate matter pollution across multiple travel modes. However, limited information is available on the comparison of in-transit exposure among cities that experience different climates and weather patterns. This study aimed to examine the variations in in-cabin particle concentrations during taxi, bus, and metro commutes among four megacities located in the inland and coastal areas of China. To this end, we employed a portable monitoring approach to measure in-transit particle concentrations and the corresponding transit conditions using spatiotemporal information. The results highlighted significant differences in in-cabin particle concentrations among the four cities, indicating that PM concentrations varied in an ascending order of, and the ratios of different-sized particle concentrations varied in a descending order of CS, SZ, GZ, and WH. Variations in in-cabin particle concentrations during bus and metro transits between cities were mainly positively associated with urban background particle concentrations. Unlike those in bus and metro transit, in-cabin PM concentrations in taxi transit were negatively associated with urban precipitation and wind speed. The variations in particle concentrations during the trip were significantly associated with passenger density, posture, the in-cabin location of investigators, and window condition, some of which showed interactive effects. Our findings suggest that improving the urban background environment is essential for reducing particulate pollution in public transport microenvironments. Moreover, optimizing the scheduling of buses and the distribution of bus stops might contribute to mitigating the in-cabin exposure levels in transit. With reference to our methods and insights, policymakers and other researchers may further explore in-transit exposure to particle pollution in different cities.

## 1. Introduction

Air pollution is recognized as one of the greatest environmental risks to public health; particulate matter (PM) is a common proxy indicator for air pollution, as it affects more people than any other pollutant [1]. Ambient particulate matter, such as PM_10_, can penetrate and lodge deep inside the lungs, and PM_2.5_ can penetrate the lung barrier and enter the blood system. Exposure to ambient fine particulate matter is strongly correlated with severe health conditions, such as respiratory and cardiovascular morbidity, cardiac ischemia, myocardial damage, and lung cancer [2,3,4,5]. A study on the Global Burden of Disease (GBD) indicated that ambient PM_2.5_ was the fifth-ranking greatest mortality risk factor in 2015, leading to 4.2 million annual deaths [6]. Given its severity, it is critical to have a better understanding of the pollutant exposure at the individual level. Pollutant exposure during commuting time may also provide useful insights [7].

Residents of Asian, European, and North American cities perform 7–10% of their daily activities in transport microenvironments (TMEs), where traffic emissions are a major pollution source [4,8]. Studies have indicated that commuters experience highly variable concentrations of ambient pollutants and face short-time extreme peak pollutant concentrations, two times higher than those at home or in urban background locations [4,9,10,11]. Commuting contributes significantly to the overall transport-related air pollution, accounting for 12–32% of the daily exposure, owing to the proximity of the individuals to the pollution source [11,12]. General public transit is of particular concern, as it transports a large share of commuters, especially in large and mega cities. Moreover, UN SDG11 indicates the goal and importance of providing access to sustainable public transport systems for all by 2030. To achieve the goal set for sustainable urban development, a comprehensive assessment of the variability in exposure concentrations in TMEs among different cities is needed to inform efficient policies in the spheres of public health and urban mobility strategies.

Various field studies have been conducted to examine exposure to particle pollution in transport microenvironments at the individual level. These studies either focused on assessing the personal exposure levels for a specific transportation mode [13] or compared exposure levels across different transport modes [14], using portable pollutant monitors deployed in TMEs or carried by investigators [2,15,16,17,18]. Moreover, several reviews have been conducted to assess and compare exposure levels in different transportation modes [2,4,9,19] and examine the air quality for a specific transport microenvironment, such as bus stations, metros, or personal vehicles [17,18,20], although there have been some inconsistencies in conclusions about differences in commuter exposure [9]. Moreover, differences in commuter exposure cannot be fully attributed to modal differences or caused by specific factors [14]. Regarding active modes, pedestrians and cyclists are exposed to lower particle concentrations when selecting routes separated from motorized traffic [19]. Regarding motorized modes, factors affecting the levels of exposure primarily relate to ventilation settings (e.g., windows open or closed and air conditioning on/off), vehicle mode (e.g., year, design, and fuel type), breathing zones with respect to the positions of passengers, doors opening at bus stops, seat positions, passenger density inside a cabin, urban air quality, and urban meteorological conditions [2,3,12,21,22,23,24].

Nevertheless, studies comparing the in-transit exposure among cities, especially cities with different climates and weather conditions, are limited. Although a small number of studies have examined in-transit exposure in different cities, these studies mainly focused on the variations in exposure between different travel modes rather than the variations in in-transit pollutants exposure among cities [25,26,27,28]. However, most of these studies only concern European and North American cities; large Asian cities with extensive public transit have been overlooked. A comprehensive and inclusive assessment of the variability in exposure concentrations experienced in TMEs is essential for formulating efficient policies on public health and urban mobility strategies across the world [29].

Within this context, the present study aims to investigate the differences in levels of exposure to inhalable PM pollution among cities during taxi, bus, and metro commuting and their associations with en-route environmental conditions. We selected four megacities, located in Central or South China, based on their urban environments and geographical positioning, ranging from coastal to inland areas, as relatively few studies have been conducted on Asian TMEs [4]. Instead of simply using trip- or time-weighted PM concentrations, we employed a portable monitoring approach based on geospatial techniques to measure in-transit PM concentrations and corresponding en-route environmental conditions with spatial and temporal information. The portable monitoring system designed in this study consists of an aerosol monitor, a temperature-relative humidity monitor, and a GPS-equipped mobile phone. This system was used to collect in-transit mass concentrations of PM_1_, PM_2.5_, and PM_10_, as well as en-route environmental conditions in terms of transit conditions, with respect to temperature and relative humidity in each city.

## 2. Materials and Methods

### 2.1. Experimental Design

**Figure 1:**
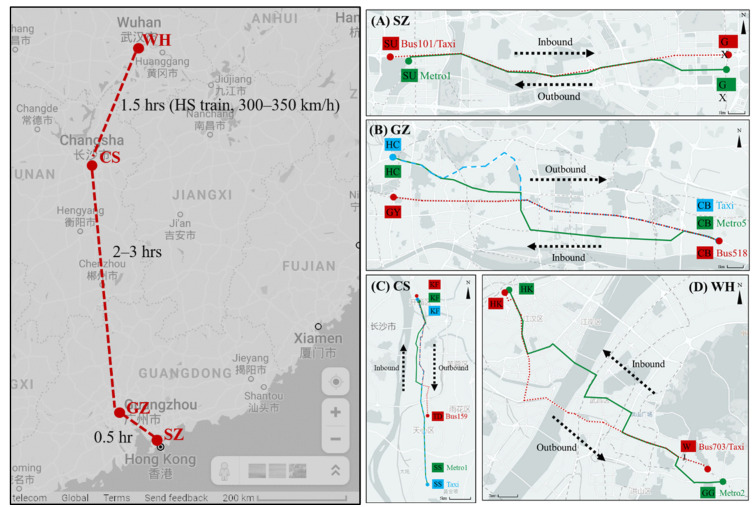
Locations of the four megacities (**left panel**) and respective survey routes (**right panel**). (**A**) survey routes of bus, metro, and taxi in SZ; (**B**) survey routes of bus, metro, and taxi in GZ; (**C**) survey routes of bus, metro, and taxi in CS; and (**D**) survey routes of bus, metro, and taxi in WH.

This study was conducted in four megacities—Wuhan (WH), Changsha (CS), Guangzhou (GZ), and Shenzhen (SZ) of China (Figure 1, left), which are key transport hubs for north–south high-speed railway network. Moreover, WH, CS, and GZ are the capital cities of Hubei, Hunan, and Guangdong Provinces, respectively, which together account for most of the Central China and South China regions, and SZ is a special economic zone of China and one of China’s super first-tier cities (i.e., Beijing, Shanghai, Guangzhou, and Shenzhen). Hunan Province borders the province-level divisions of Hubei to the north and Guangdong to the south. As of 2021, WH is the most populous city in Central China, housing a population of 13.65 million within urban built-up area of 1200 km^2^. CS is the largest city of Hunan Province and home to 10.06 million people within an urban built-up area of 560.80 km^2^. GZ is the largest city of Guangdong province and home to 18.81 million people within an urban built-up area of 1351 km^2^. SZ borders Hong Kong to the south and is home to 17.63 million people within an urban built-up area of 1217 km^2^. WH and CS are inland cities, whereas GZ and SZ are coastal cities; the winters in SZ and GZ are mild and relatively dry, whereas in CS and WH, winters are much colder.

A field survey route for each city was designed based on the rule that it must travel along a major boulevard, goes through the central area of the city, and features a large volume of passengers (Figure 1, right panel). Given that ridership on metros is higher than that on buses, we first selected a metro route that ran through the center of different urban districts; then, we selected bus and taxi routes to show the similar trajectory for comparisons among transport modes. For WH, we selected metro line 2, which connects the central locations of the Hankou and Wuchang districts, connecting the east and west. For CS, we selected metro line 1, which connects the central locations of the Kaifu, Furong, Tianxin, and Yuhua districts, connecting the north and south. For GZ, we selected metro line 5, which runs through the central locations of the Liwan, Yuexiu, Tianhe, and Huangpu districts, connecting the east and west. Finally, for SZ, we selected metro line 1, which passes through the central locations of the Baoan, Nanshan, Futian, and Luohu districts.

**Figure 2:**
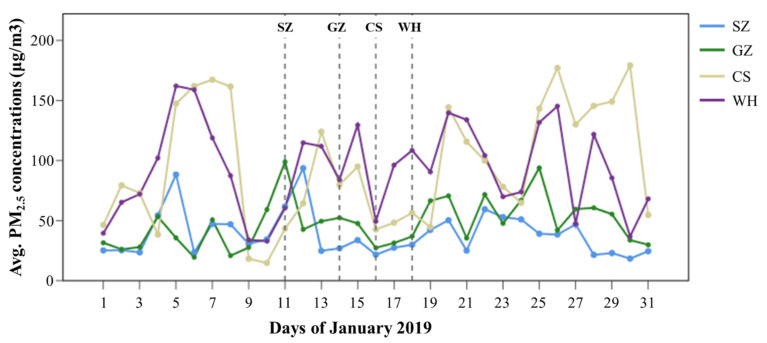
Urban ambient PM_2.5_ concentrations measured by fixed urban monitoring stations in the four cities in January 2019 (7:00–20:00).

According to the official data of PM_2.5_ concentrations from fixed urban monitoring stations in January, each of the four cities featured different urban ambient conditions. The average PM_2.5_ concentrations from selected urban monitoring stations near the survey route of each city is shown in Figure 2. In general, urban PM_2.5_ concentrations increase from southern cities to northern cities (i.e., SZ < GZ < CS < WH), with a few exceptions during the month. The field survey was conducted in SZ (11 January), GZ (14 January), CS (16 January), and WH (18 January) during the peak travel hours on weekdays in 2019. Compared to the air quality on rest of January in each city, the air quality on survey day in CS was better than those on most days of January, which is opposite to the findings of GZ and SZ, and the air quality on survey day in WH showed an average level of January. The PM_2.5_ concentrations on survey days of the four cities have exceeded the limits of PM_2.5_ concentrations in terms of 24-h mean defined by WHO (i.e., 15 μg/m^3^) and China (i.e., Class1-35 μg/m^3^, and Class2-75 μg/m^3^). The three transportation modes (taxi, bus, and metro) were quasi-simultaneously surveyed along selected inbound–outbound routes. Data on particle concentrations, temperature, relative humidity, and transit conditions were recorded while using taxis, public buses, and metros in the morning and evening peak hours.

### 2.2. Mobile-Based In-Transit Measurements

We designed a mobile-based monitoring approach to perform simultaneous measurements of PM concentrations and en-route environmental conditions in terms of temperature, relative humidity, and transit conditions during the trip. Such a monitoring approach was conducted by investigators using a set of three devices, i.e., a mobile phone (Android system), an aerosol monitor, and a temperature-relative humidity monitor. The mobile phone was equipped with a self-designed field survey application (app) that was primarily used for logging the transit conditions of the bus and metro routes; it contains a GPS function. The three devices used are lightweight and portable. To ensure simultaneous measurements using the three devices, the times (y-m-d hh:mm:ss) of these devices were synchronized before each survey. For the measurement of each transit, each of three investigators carried a set of devices when riding one mode of transportation. GPS data, PM concentrations, temperature, and relative humidity were automatically logged by devices, but the transit conditions of the bus and metro were logged using our app (the fields are outlined in Table S1). Regarding taxis that go directly from one location to another, the in-cabin transit conditions mainly include ventilation conditions. In our study, to reflect the actual conditions of travelling via taxi, the ventilation conditions were manipulated by taxi drivers based on their habits, and one of the two investigators riding in the taxi recorded temporal changes in ventilation conditions during the trip.

Additionally, we used DustTrak DRX Aerosol Monitor 8534 (DustTrak DRX8534, TSI Inc., Shoreview, MN, USA), which is an advanced version of the DustTrak Aerosol Monitors. The scientific principle of this device and the performance in real time are clearly explained in related literature [30,31,32,33]. To ensure the accuracy of each measurement, we performed zero calibration for the monitor using a high efficiency particulate air (HEPA) zero-filter assembly before each use. The data logging interval was set to 1 s, the flow point to 3.0 L/min, and the photometric and size calibration factors to 0.38 and 1.0, respectively, following the suggestions from the monitor manufacturer and related literature given that our focus is variations in PM concentrations and relative concentrations [31,33,34].

We used Elitech RC-4HA (Model Elitech RC-4HA, Jingchuang Electronics Co., Ltd., Xuzhou, China), which is a real-time and handy monitor for automatic measurement, to measure temperature and relative humidity. This equipment has been certified by the US FDA with tools of the Good Storage Practices (GSP) standard, which have been widely used in the field of logistics, especially for warehouses and cold chains. The temperature and humidity measurements ranged from −30 °C to +60 °C and 0–99% RH, respectively.

For the measurement of transit conditions using our designed mobile app, the app fields (Table S1) were logged by the investigators during the trip and a time stamp (yy-mm-dd hh:mm:ss) was automatically generated for each record. In terms of identifying the periods of idling (door-open) and moving (door-closed) for each route segment, we formulated a strict rule to ensure that every investigator followed the same procedure in field survey when logging the app field called “location on route segment”. Specifically, the investigator tapped “location 1” when the bus/metro door closed and the vehicle left the bus/metro platform, tapped “location 2” when the bus/metro approached the middle of a route segment, and tapped “location 3” when the bus/metro arrived at a bus stop/metro station before the door opened. Consequently, each route segment between two consecutive stops/stations could be divided into two subsegments: the moving period and idling period.

### 2.3. Quality Check and Validation of Measurements

We performed zero calibration for each aerosol monitor before each measurement, using a HEPA zero filter assembly, to ensure measurement accuracy. Three DRX8534 monitors were collocated, and simultaneous measurements were conducted to observe whether the monitor displayed the same readings every day before the field survey. Some studies have reported that humidity affects the overestimation of basic DustTrak models [35,36]; however, no association between humidity and real-time DRX8534 measurements (advanced version) has been reported [33,34]. To eliminate the discrepancy due to the impact of humidity on the readings of aerosol monitors, the real-time temperature and relative humidity were observed in addition to those recorded by the DRX8534 monitor during each survey, given that some studies reported that relative humidity of 80% and above can affect the readings of optical monitors [37,38]. In addition, to ensure the stability of measurements, we also compared the PM_2.5_ and PM_10_ concentrations measured by investigators in transit with those measured by official fixed urban monitoring stations throughout the measurement period.

### 2.4. Data Collection and Preprocessing

#### 2.4.1. Data Collection

On each sampling day, data were collected from 12 transits for each city. Out of 12 transits, four were made for each of the three transport modes: public buses, taxis, and metros. The details of the data collected (such as data attributes, data source, and data format) in this study are described in Table S2. In addition to the self-measured data (PM_1_, PM_2.5_, and PM_10_ concentrations, transit conditions, temperature, and relative humidity), data on hourly value of urban ambient PM_2.5_ and PM_10_ concentrations were obtained from the official website of Ministry of Ecology and Environment (https://air.cnemc.cn:18014/ accessed on 10 February 2019); data on daily value of urban meteorological conditions (temperature, relative humidity, precipitation, and wind speed) were provided by the Resource and Environment Science and Data Center (https://www.resdc.cn/ accessed on 10 February 2019).

#### 2.4.2. Data Preprocessing

The measured data of PM_1_ concentrations included abnormal values, i.e., 97 measurements (0.07% of the total), some of which were collected outside the cabins and had negative particle values. A study [39] reported that the negative values logged by DRX8534, which might be attributed to the sudden movement of the monitor or the airflow between the indoor and outdoor environments, should be removed from the dataset. In this study, we removed the negative values from dataset because they may indicate the sudden movement of the monitor, likely caused by the running conditions of vehicles or the movement of the passengers preparing to enter or exit at the next stop/station.

The data collected from different monitors, i.e., aerosol monitors, temperature and relative humidity monitors, and app, were integrated based on the time stamps. The timestamp served as a temporal reference for linking each data point of particle concentration, app record, temperature and humidity values, urban ambient PM concentrations, and meteorological conditions. In this manner, each parameter of PM concentration data (i.e., PM_1_, PM_2.5_, and PM_10_ concentrations measured at timestamp “dd/mm/year, hh:mm:ss”) was labeled with the respective en-route environmental conditions: self-measured temperature and relative humidity and the spatial information (e.g., transit conditions and GPS coordinates) as well as urban ambient PM concentrations and urban meteorological conditions.

### 2.5. Data Analysis

We performed statistical analyses to examine the variations in in-cabin PM concentrations between the four cities and their associations with en-route environmental conditions. All data analyses and visualizations were performed using the SPSS 26.0 (IBM Corp., Armonk, NY, USA) and ArcGIS 10.6 (Esri, Redlands, CA, USA) software.

To compare the variations in in-cabin PM concentrations among cities, we first computed the minimum, mean, maximum, and percentile (including 19 data points, i.e., 5th, 10th, 15th, 20th…90th, and 95th) values for each trip, in an effort to ensure a reliable comparison among cities, as the trip durations were not equally long, which would have created an inconsistency in the number of data points obtained. We then employed Kruskal–Wallis (K–W) test to examine the statistical differences in in-cabin PM concentrations and different particle size fractions among cities, because the data did not follow a normal distribution. Moreover, the Jonckheere–Terpstra (J–T) test was used to investigate the trend in variations of in-cabin PM concentrations among cities. The J–T test indicates whether the in-cabin PM concentrations of the four cities ascend or descend in the assumed order. Based on the average urban ambient PM_2.5_ concentrations measured by the fixed urban monitoring stations on survey day (Figure 2), the ascending order of PM concentrations of the four cities was assumed to be CS, SZ, GZ, and WH for the J–T test.

To understand the associations between en-route environmental conditions and the variation in PM concentrations in transit, a set of en-route environmental factors inside and outside the cabin was defined in this study and is explained in Table 1. In order to identify the factors that best explained variations in in-transit particle concentrations, specific statistical analyses were conducted as follows:

(1) Effects of in-cabin environmental conditions (for bus and metro): in-cabin environmental conditions might vary across route segments during the trip; therefore, we investigated the effect of in-cabin environmental conditions on the variations in PM concentrations between route segments during the trip. Given that the PM concentrations data of some trips were not normally distributed, we selected trips that showed normal distribution of PM concentration data. For each trip, we performed a factorial ANCOVA analysis to examine the effect of in-cabin transit conditions on the variations in PM concentrations between route segments, wherein travel duration was considered a covariate. Moreover, factorial ANOVA analysis was conducted when no significant effect of the covariate was identified. Additionally, we defined the data of PM concentrations measured during the moving period of route segments as the dependent variable, considering that the PM concentrations measured during the idling period might be affected by other extraneous factors, such as indoor–outdoor air/gas exchange when passengers were boarding and alighting.
(2) Effects of in-cabin environmental conditions (for taxi): the in-cabin environmental conditions of taxis included in-cabin ventilation conditions, temperature, and relative humidity. The ventilation conditions of taxis were manipulated by taxi drivers based on their habits and, thus, varied. Therefore, we employed simplified descriptive statistics to examine whether the changes in PM concentrations were associated with the changes in ventilation conditions, i.e., comparing the average PM concentrations measured during the five minutes before and after the change in ventilation conditions.
(3) Effects of out-cabin and urban background environmental conditions: For the effect of out-cabin environments measured by our investigators when waiting for buses and metro trains, we examined the correlations between the in-cabin PM concentrations measured on first route segment (moving period) and the PM concentrations measured at each bus platform and metro station before boarding, which indicated whether the out-cabin PM concentrations during the idling period had any effect on the in-cabin PM concentrations. Regarding the effect of urban background environment, which aims for cities’ comparison, we examined the correlation between in-cabin PM concentrations measured during the moving periods in each of the four cities and their urban background PM concentrations, as well as meteorological conditions.

## 3. Results and Discussion

### 3.1. General Description on Exposure Levels and Transit Conditions

**Figure 3:**
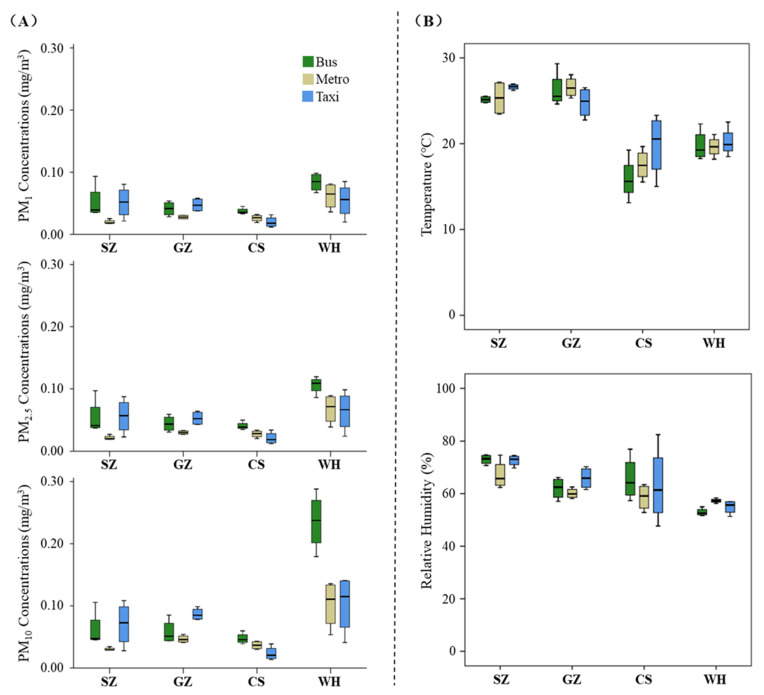
Distribution of in-cabin PM concentrations (**A**) and temperature and relative humidity (**B**) of each transit by City and Mode of transportation.

Figure 3A describes the distribution of in-cabin average PM_1_, PM_2.5_, and PM_10_ concentrations of each transit by city and travel mode (the detailed measurements on each transit are outlined in Table S3). In general, the in-cabin average PM concentrations measured in WH were higher than those in the other three cities. The in-cabin average PM concentrations measured on the metros and buses of CS were quite similar to those of SZ and GZ, while the in-cabin average PM concentrations measured in Taxi’s were lower than those of SZ and GZ. This may be attributed to the fact that the CS showed quite low urban ambient particle concentrations on the survey day on which it was snowing during the evening transit, although the urban ambient conditions in CS were not as clear as those in SZ and GZ on most days of the month (Figure 2). In addition, when comparing the average in-cabin PM concentrations among three travel modes, different findings were identified for the four cities. Specifically, in SZ and GZ, metro trips had the lowest average PM concentrations, while taxi trips had the highest average PM concentrations; in CS and WH, taxi trips had the lowest average PM concentrations, and buses had the highest average PM concentrations.

Figure 3B describes the distribution of in-cabin average temperature and relative humidity of each transit by city and travel mode. In addition, the in-cabin temperature indicated two clusters, i.e., coastal cities (SZ and GZ) and inland cities (CS and WH), characterized by different weather conditions. The in-cabin relative humidity was higher in SZ than in the other three cities, but it was comparable with that of its coastal counterpart, GZ. It should be noted that the heating systems inside the cabins of the buses and metros in CS and WH were turned on during the winter season (January), but the ventilation conditions inside the taxis were manipulated by the driver, which will be discussed in detail in Section 3.3.

**Figure 4:**
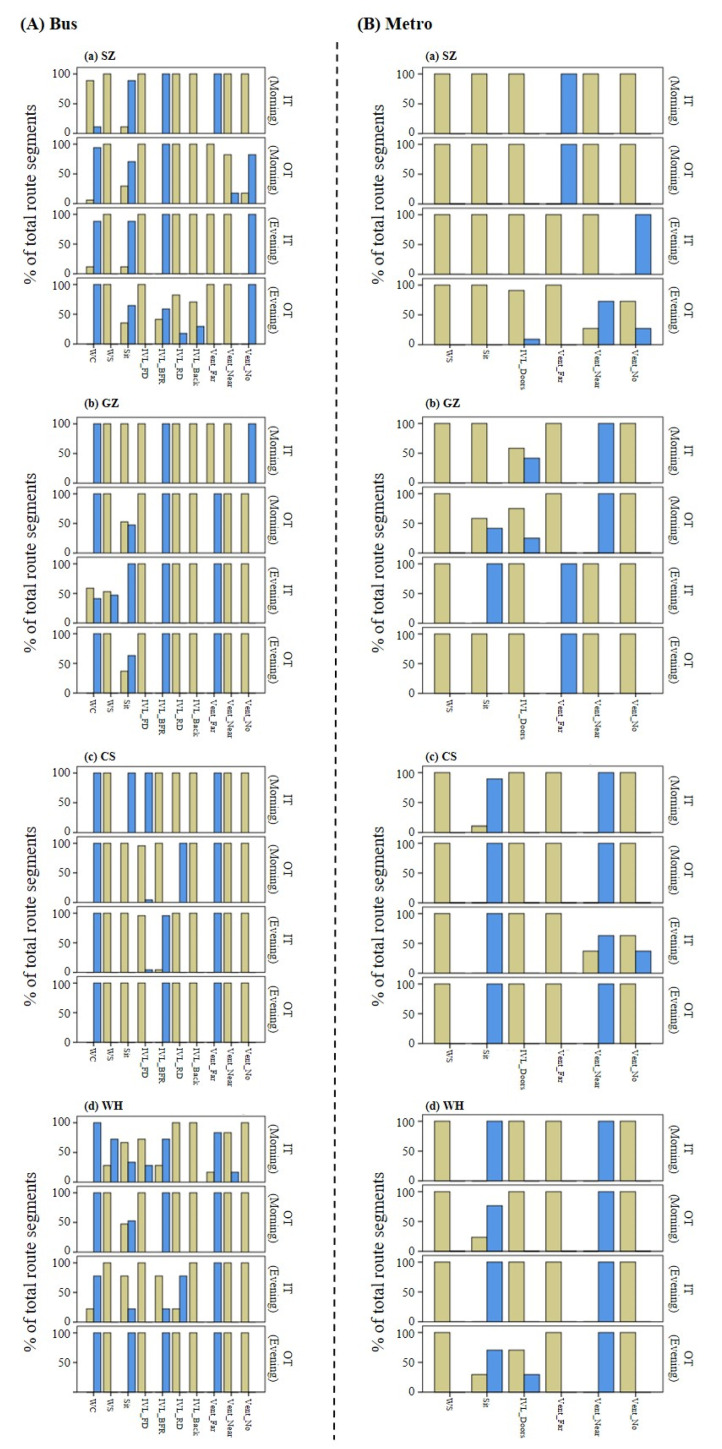
Percentage of bus route segments (**A**) and metro route segments (**B**) within each category of in-cabin transit conditions during a trip. (**a**–**d**) represent the city of SZ, GZ, CS, and WH, respectively.

Figure 4 describes the percentage of route segments within each category of in-cabin transit conditions during a given trip. In total, 12 bus trips and 4 metro trips were characterized by changes in transit conditions between route segments during the trip. In both bus and metro trips, it appeared that changes in transit conditions were mostly influenced by investigator posture (sit or stand), window condition (closed or open), the location of investigator inside the cabin, and passenger density.

### 3.2. Variations in In-Transit Exposure Levels among Cities

For each trip, 22 statistical values (min, mean, max, and percentiles) of PM concentrations were generated to ensure a reliable comparison among cities, because the duration of each trip was not comparable.

Table 2 describes the statistical results of differences in the in-cabin PM concentrations among the four cities, and Table 3 describes the statistical results of differences in the ratios of in-cabin PM concentrations among the four cities. For each of three transportation modes, significant differences were observed in the PM concentrations (Table 2) and ratios of different-sized particle concentrations (Table 3) among the four cities. Moreover, the J–T test revealed a significant trend: in-cabin PM concentrations of taxi, bus, and metro trips ascended in the city order of CS→SZ→GZ→WH, except for those measured during inbound taxi trips in the evening. The ratios of PM_1_/PM_2.5_, PM_1_/PM_10_, and PM_2.5_/PM_10_ concentrations of taxi, bus, and metro trips descended in the order CS→SZ→GZ→WH. This indicates that the PM concentrations increased, but the proportion of small particles (PM_1_ and PM_2.5_) decreased in the city order of: CS→SZ→GZ→WH.

### 3.3. Effects of En-Route Environmental Conditions on the Variation in Exposure Levels

The description of en-route environmental factors and the approaches for examining the effects of en-route environmental conditions are outlined in detail in Section 2.5.

#### 3.3.1. In-Cabin Environmental Conditions

**Figure 5:**
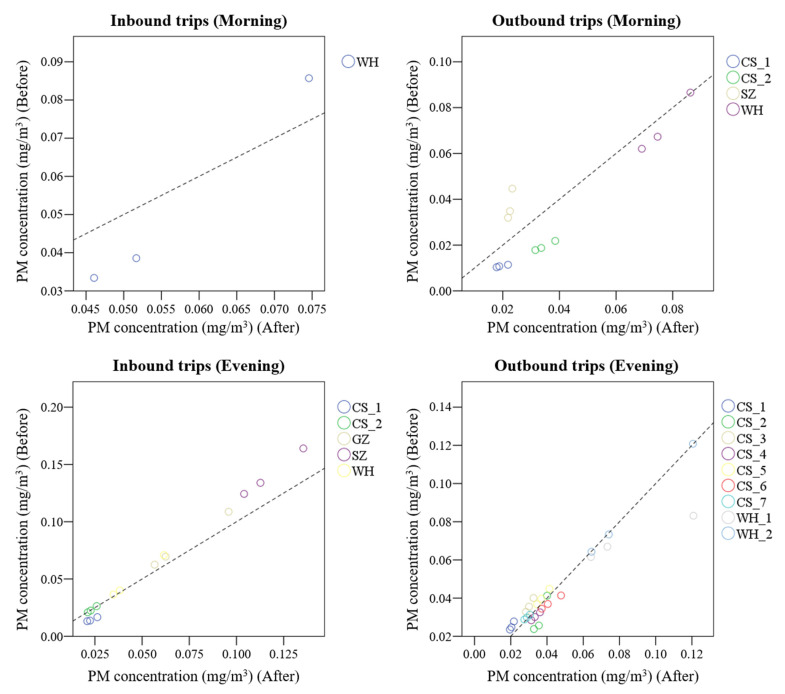
Comparison of particulate matter concentrations before and after changing ventilation conditions.

Referring to the in-cabin transit conditions of the taxis, including ventilation conditions, temperature, and relative humidity inside the cabin, Figure 5 shows a comparison of the average PM concentrations measured in the five minutes before and after a change in ventilation conditions (Table 4). The results showed that PM concentrations increased when the windows were opened but decreased if the air conditioner was turned on. Moreover, PM concentrations did not decrease if only one window was left open, the rest were closed, and the air conditioner was kept off. However, PM concentrations decreased when the window nearest to the investigator (back seat) was closed and the front windows were kept open. This may be attributed to air circulation through the front windows, leading to the movement of PM out of the cabin, particularly from the back seat. No significant correlation was observed between the in-cabin temperature and relative humidity and the variations of in-cabin PM concentrations among cities.

For determining the effect of in-cabin travel environment conditions on the variations in PM concentrations between route segments during the trip, we selected the trips that showed a variation in the in-cabin transit conditions (see Figure 4) as well as a normal distribution of in-cabin PM concentrations to conduct factorial analyses. Consequently, seven bus trips and four metro trips were selected (see Table S4). Table 5 describes the results of the factorial analysis, i.e., the factors that significantly contribute to the variation in PM concentrations during the trip and the variability explained by those factors (details of statistical results are shown in Table S5). In general, for both bus and metro transit, passenger density was positively correlated with the variation in PM concentrations during the trip, PM concentrations were higher when investigators were sitting compared to those when standing, and PM concentrations were significantly higher when investigators stood near the vents. When the bus cabin was crowded, PM_10_ concentration was significantly higher when windows were closed compared to that when windows were opened. Moreover, PM concentrations were higher when investigators were standing closer to the doors than when staying in other areas of the bus cabin, while PM concentrations were lower when investigators stood near the doors compared than when staying in the seating area. Travel duration of the route segments show a negative effect on PM concentrations.

#### 3.3.2. Out-Cabin and Urban Background Environmental Conditions

Table 6 describes the correlation coefficients between in-cabin PM concentrations measured during the moving period and out-cabin and urban background environmental conditions compared by city. In general, in-cabin PM concentrations measured on the first route segment of bus and metro trips were significantly positively correlated with those measured at the bus and metro platforms, from metro turnstile to platform, and from metro entrance to turnstile. Regarding the urban background environment, for bus and metro transit, the variations in in-cabin PM concentrations among cities were significantly positively associated with their urban background PM_10_ concentrations. For urban meteorological conditions, urban precipitation and wind speed were significantly negatively associated with the variation in in-cabin PM concentrations of only taxi transits among cities, possibly owing to the fact that bus windows were mainly closed and metro trains travel underground, where exposure to urban meteorological effects is limited.

## 4. Discussion

The results of this study highlight significant differences in the in-cabin PM concentrations and ratios of different-sized particle concentrations among the four cities, i.e., the variations of in-cabin PM concentrations among cities were ranked in an order: CS < SZ < GZ < WH, and the variations in the ratios of PM_1_/PM_2.5_, PM_1_/PM_10_, and PM_2.5_/PM_10_ among cities were ranked in an order: CS > SZ > GZ > WH. This inverse relationship indicates that the overall PM concentrations increased, but the proportion of small particles (PM_1_ and PM_2.5_) decreased from southern (coastal) to northern (inland) cities. The variations in the in-cabin PM concentrations among cities were significantly positively associated with the urban background PM_10_ concentrations. Although public buses are powered by electricity in SZ, the in-cabin PM concentrations of bus trips in SZ were higher than those in CS on survey day. This implies that improving the urban background environment is a crucial factor for reducing particulate pollution and human exposure levels in transport microenvironments.

When comparing the exposure levels of the in-cabin PM pollution among buses, metros, and taxis, the findings in the four cities indicated two dominant trends: SZ and GZ had the lowest PM concentrations inside metro cabins and the highest PM concentrations inside taxi cabins, while CS and WH had the lowest PM concentrations inside taxi cabins and the highest PM concentrations inside bus cabins, which might be attributed to the fact that SZ and GZ are considered ‘super first-tier cities’, in that their governments invest in public transport: for example, buses are mainly powered by electricity in SZ and GZ, so commuters using the bus and the metro have a lower exposure to particle pollution than riding in the taxi.

Regarding the effect of in-cabin transit conditions on the variations of PM concentrations during the trip, passenger density showed a positive effect on the exposure levels on the buses and metros [40]. PM concentrations were higher when investigators were sitting as opposed to standing inside bus and metro cabins, which might be because the cabins can be quite crowded in peak hours, and the heights of the seats are quite low; thus, the movement of passengers easily affects the movement of particles in the lower areas of the cabin [19]. PM concentrations were also higher when investigators stood near a vent inside bus and metro cabins, which may be attributed to the indoor–outdoor air exchange. Standing near the rear doors of buses also has a negative impact on the in-cabin exposure level, attributing to passenger movement [41]. Moreover, out-cabin PM concentrations measured during idling periods have a negative effect on the PM concentrations inside bus and metro cabins, and the travel duration of route segments shows a negative effect on PM concentrations inside bus cabins [23,26]. This implies that frequently opened doors have a negative impact on the PM concentrations inside the cabin, possibly owing to the air exchange between the indoors and outdoors and passenger movements (boarding and alighting). This suggests that optimizing the distribution of bus stops can mitigate the in-cabin exposure levels in transit. For taxis, in-cabin PM concentrations increased when the windows were open but decreased if the air conditioner was on [42]. Moreover, PM concentrations decreased when the window nearest to the investigator (back seat) was closed and the front windows were kept open, which may be attributed to air circulation through the front windows. In addition, in-cabin PM concentrations of taxis were significantly negatively associated with urban wind speed and precipitation, which might be attributed to the higher precipitation and wind speed contributing to the dilution of particle concentrations.

This study has some limitations. For example, the measurements were conducted only during commuting periods on one day in each city, due to that we have limited time and resources of portable monitors and human labor. Additional measurements should be conducted to verify the variations in in-transit exposure among cities with different air quality, located in different regions, and to investigate particle pollution trends during different times and seasons. Additionally, future studies could benefit from testing various controlled ventilation settings inside the cabin and urban PM concentrations. Nevertheless, we believe that this study provides novel insights into the variations in in-transit PM concentrations among major Chinese cities in South and Central China, as well as the associations among transit conditions, urban meteorological conditions, and urban air quality, which can be achieved using spatial data and a multi-sourced dataset. With reference to these methods and insights, the results of this study may help policymakers and other researchers explore a new legislation or policy regarding in-transit exposure to particle pollution in different cities to improve the environment of public transit and work toward the sustainable development of cities. Combined with studies conducted in the cities in North America and Europe, the results of this and similar studies can provide a more holistic understanding of air pollution dynamics globally.

## 5. Conclusions

Few studies have compared the in-transit inhalable PM exposure among cities (especially in Asian TMEs) using spatial techniques while measuring and examining exposure to particulate pollution in transit. To address this research gap, we proposed a mobile-based monitoring approach to investigate the variations in in-cabin PM concentrations during taxi, public bus, and metro commutes in four megacities of China: SZ, GZ, CS, and WH, which include coastal and inland cities characterized by different urban weather conditions. Findings from our study suggested that improving the urban background environment is essential for reducing particulate pollution in public transport microenvironments. Moreover, optimizing the scheduling of buses and the distribution of bus stops might contribute to mitigating the in-cabin exposure levels in transit.

## Figures and Tables

**Table 1:** Description of en-route environmental factors.

Factor	Type	Factor Name	Factor Description
**In-Cabin**	Transit Condition	Window Closed (WC)	dummy variable (0 or 1), “1” = “Windows surrounding the investigator are closed inside the bus cabin”
		Sit	dummy variable (0 or 1), 1 indicate “The investigator was sitting when conducting measurement inside the cabin”
		Ventilator_Near (VN)	dummy variable (0 or 1), 1 indicate “Vents are located directly above or below the investigator”
		Ventilator_Far (VF)	dummy variable (0 or 1), 1 indicate “Vents are surrounding the investigator but are not located directly above or below the investigator”
		Ventilator_No (VO)	dummy variable (0 or 1), 1 indicate “No vent is in the area surrounding the investigator”
		In-Vehicle Location_FrontDoor (IVL_FD)	dummy variable (0 or 1), 1 indicate “The position of the investigator inside the bus cabin is near the front door”
		In-Vehicle Location_BFR (IVL_BFR)	dummy variable (0 or 1), 1 indicate “The position of the investigator inside the bus cabin o is between the front and rear doors”
		In-Vehicle Location_RearDoor (IVL_RD)	dummy variable (0 or 1), 1 indicate “The position of the investigator inside the bus cabin is near the rear door”
		In-Vehicle Location_Back (IVL_Back)	dummy variable (0 or 1), 1 indicate “The position of the investigator inside bus cabin is at the back part”
		In-Vehicle Location_Seats (IVL_Seats)	dummy variable (0 or 1), 1 indicate “The position of the investigator inside the metro cabin is the seating area”
		In-Vehicle Location_Doors (IVL_Doors)	dummy variable (0 or 1), 1 indicate “The position of the investigator inside the metro cabin is near the door”
		Passengers’ density (PD)	The density of passengers inside the cabin (value = 1, 2, 3, 4, 5).
		Trip duration (TD)	The travel time (minutes) of each trip.
	Tem	Temperature (Tem)	The temperature inside the cabin measured by the investigator.
	RH	Relative Humidity (RH)	The relative humidity inside the cabin measured by the investigator.
**Out-Cabin**	Out-cabin PM	PM concentrations (Bus platform)	PM_1_, PM_2.5_, and PM_10_ concentrations measured at bus platform while investigator was waiting for the bus.
		PM concentrations (Metro station)	PM_1_, PM_2.5_, and PM_10_ concentrations measured by investigator while walking from entrance to platform, we divided this period into three parts: E-T represents walking from entrance to turnstile, T-P represents walking from turnstile to platform, and Platform represents the period waiting for the train.
	Urban background PM	Urban ambient PM concentration	PM_2.5_, PM_10_ concentrations measured by fixed urban PM monitoring stations.
	Urban Meteorological Condition	Temperature (U-Tem)	min, mean, max values of temperature measured by fixed urban meteorological stations (unit 0.1 °C).
		Relative Humidity (U-RH)	min, mean, max values of relative humidity measured by fixed urban meteorological stations (unit 1%).
		Precipitation (PRE)	mean values of precipitations measured by fixed urban meteorological stations, two groups (i.e., 8–20 for day, 20–8 for night) (unit 0.1 mm).
		Wind Speed	mean and max values of wind speed measured by fixed urban meteorological stations (unit 0.1 m/s).

**Table 2:** Kruskal–Wallis (K–W) and Jonckheere–Terpstra (J–T) tests for variations in in-cabin particulate matter concentrations among cities for each travel mode. (a) Kruskal–Wallis Test Statistics. (b) Jonckheere–Terpstra Test Statistics.

(**a**)								
			**Morning**			**Evening**		
			**PM_1_**	**PM_2.5_**	**PM_10_**	**PM_1_**	**PM_2.5_**	**PM_10_**
**Taxi**	**IT ^a^**	Chi-Square	47.654	46.413	52.191	50.499	50.295	48.241
		Monte Carlo Sig. *	0.000	0.000	0.000	0.000	0.000	0.000
	**OT ^b^**	Chi-Square	57.375	65.370	61.915	37.577	43.235	52.554
		Monte Carlo Sig. *	0.000	0.000	0.000	0.000	0.000	0.000
**Bus**	**IT**	Chi-Square	40.933	49.289	46.470	42.543	44.964	48.080
		Monte Carlo Sig. *	0.000	0.000	0.000	0.000	0.000	0.000
	**OT**	Chi-Square	54.147	54.151	41.488	36.378	42.317	43.687
		Monte Carlo Sig. *	0.000	0.000	0.000	0.000	0.000	0.000
**Metro**	**IT**	Chi-Square	55.508	53.758	51.148	35.282	37.080	26.882
		Monte Carlo Sig. *	0.000	0.000	0.000	0.000	0.000	0.000
	**OT**	Chi-Square	51.782	51.929	46.490	61.612	63.139	48.983
		Monte Carlo Sig. *	0.000	0.000	0.000	0.000	0.000	0.000
(**b**)								
			**Morning**			**Evening**		
			**PM_1_**	**PM_2.5_**	**PM_10_**	**PM_1_**	**PM_2.5_**	**PM_10_**
**Taxi**	**IT**	Std.J–T Statistics	4.892	5.813	7.449	−0.518	−0.149	0.966
		Monte Carlo Sig.	0.000 *	0.000 *	0.000 *	0.305	0.434	0.171
	**OT**	Std. J–T Statistics	8.855	9.362	9.005	5.124	5.791	7.337
		Monte Carlo Sig. *	0.000	0.000	0.000	0.000	0.000	0.000
**Bus**	**IT**	Std.J–T Statistics	6.059	6.565	6.292	3.393	4.072	6.256
		Monte Carlo Sig. *	0.000	0.000	0.000	0.000	0.000	0.000
	**OT**	Std. J–T Statistics	2.635	2.635	5.176	5.687	5.530	5.149
		Monte Carlo Sig.	0.004	0.004	0.000 *	0.000 *	0.000 *	0.000 *
**Metro**	**IT**	Std. J–T Statistics	5.028	5.549	6.817	3.224	3.368	3.517
		Monte Carlo Sig. *	0.000	0.000	0.000	0.000	0.000	0.000
	**OT**	Std. J–T Statistics	6.241	6.724	6.682	6.199	6.408	5.809
		Monte Carlo Sig. *	0.000	0.000	0.000	0.000	0.000	0.000

^a^ IT: Inbound Trip; ^b^ OT: Outbound Trip. * 99% CI (Lower Bound~Upper Bound): 0.000~0.000.

**Table 3:** Kruskal–Wallis (K–W) and Jonckheere–Terpstra (J–T) tests for variations in ratios of in-cabin particulate matter concentrations among cities for each travel mode. (a) Kruskal–Wallis Test Statistics. (b) Jonckheere–Terpstra Test Statistics.

(**a**)								
			**Morning**			**Evening**		
			**PM_1_/PM_2.5_**	**PM_1_/PM_10_**	**PM_2.5_/PM_10_**	**PM_1_/PM_2.5_**	**PM_1_/PM_10_**	**PM_2.5_/PM_10_**
**Taxi**	**IT ^a^**	Chi-Square	49.345	44.320	39.665	39.837	44.615	41.997
		Monte Carlo Sig. *	0.000	0.000	0.000	0.000	0.000	0.000
	**OT ^b^**	Chi-Square	42.507	44.258	41.048	45.203	48.507	45.682
		Monte Carlo Sig. *	0.000	0.000	0.000	0.000	0.000	0.000
**Bus**	**IT**	Chi-Square	41.019	38.381	36.120	50.921	54.507	53.130
		Monte Carlo Sig. *	0.000	0.000	0.000	0.000	0.000	0.000
	**OT**	Chi-Square	30.795	28.086	26.933	57.334	46.640	43.020
		Monte Carlo Sig. *	0.000	0.000	0.000	0.000	0.000	0.000
**Metro**	**IT**	Chi-Square	22.695	26.464	24.234	5.863	7.764	6.631
		Monte Carlo Sig.	0.000 *	0.000 *	0.000 *	0.120	0.053	0.085
	**OT**	Chi-Square	12.341	10.606	9.764	21.889	14.544	11.731
		Monte Carlo Sig.	0.005	0.012	0.017	0.000 *	0.002	0.005
(**b**)								
			**Morning**			**Evening**		
			**PM_1_/PM_2.5_**	**PM_1_/PM_10_**	**PM_2.5_/PM_10_**	**PM_1_/PM_2.5_**	**PM_1_/PM_10_**	**PM_2.5_/PM_10_**
**Taxi**	**IT**	Std.J–T Statistics	−7.300	−5.947	−5.318	−6.901	−7.252	−6.798
		Monte Carlo Sig. *	0.000	0.000	0.000	0.000	0.000	0.000
	**OT**	Std. J–T Statistics	−6.155	−5.040	−4.528	−6.025	−7.758	−7.501
		Monte Carlo Sig. *	0.000	0.000	0.000	0.000	0.000	0.000
**Bus**	**IT**	Std.J–T Statistics	−4.364	−5.309	−5.321	−4.772	−5.719	−5.902
		Monte Carlo Sig. *	0.000	0.000	0.000	0.000	0.000	0.000
	**OT**	Std. J–T Statistics	−4.020	−5.320	−5.283	−2.069	−3.616	−4.267
		Monte Carlo Sig.	0.000 *	0.000 *	0.000 *	0.018	0.000 *	0.000 *
**Metro**	**IT**	Std. J–T Statistics	−4.152	−4.615	−4.422	−1.891	−2.591	−2.434
		Monte Carlo Sig.	0.000 *	0.000 *	0.000 *	0.031	0.005	0.008
	**OT**	Std. J–T Statistics	−2.208	−2.811	−2.561	−3.849	−2.673	−2.237
		Monte Carlo Sig.	0.014	0.003	0.005	0.000 *	0.004	0.012

^a^ IT: Inbound Trip; ^b^ OT: Outbound Trip. * 99% CI (Lower Bound~Upper Bound): 0.000~0.000.

**Table 4:** Description of ventilation conditions before and after the changes when commuting in a taxi.

		Before (Changing Ventilation)	After (Changing Ventilation)	Differences (After–Before)		
				PM_1_	PM_2.5_	PM_10_
IT (Morning)	WH	WC ^a^ (Heating)	WO ^b^ (driver)	0.013	0.013	−0.011
OT(Morning)	CS_1	WC	WO (1/5-driver)	0.007	0.008	0.010
	CS_2	WO (1/5-driver)	WC	0.014	0.015	0.017
	SZ	WO	AO ^c^	−0.010	−0.012	−0.021
	WH	WC	WO (small-front passenger seat)	0.007	0.007	−0.0003
IT (Evening)	CS_1	AO (Heating) and WC	WO (front windows)	0.008	0.009	0.010
	CS_2	WO (front windows)	AO (Heating) and WC	0.0003	0.0003	−0.0004
	SZ	WO (front windows)	WC (1/3-front windows)	−0.020	−0.021	−0.029
	GZ	WO	WC (Back windows)	−0.006	−0.007	−0.013
	WH	WC	WO (small-front windows)	−0.002	−0.002	−0.010
OT(Evening)	CS_1	WC and AO (Heating)	WO (1/3-driver) and AO (Heating)	−0.004	−0.004	−0.006
	CS_2	WO (1/3-driver) and AO (Heating)	WO (1/2-driver) and AO (Heating)	0.009	0.010	−0.001
	CS_3	WO (1/2-driver) and AO (Heating)	WC and AO (Heating)	−0.004	−0.005	−0.007
	CS_4	WC and AO (Heating)	WO (1/3-driver) and AO (Heating)	0.003	0.003	0.004
	CS_5	WO (1/3-driver) and AO (Heating)	WO (driver) and AO (Heating)	−0.002	−0.003	−0.004
	CS_6	WO (driver) and AO (Heating)	WC and AO (Heating)	0.003	0.004	0.006
	CS_7	WC and AO (Heating)	WO (1/2-driver) and AO (Heating)	−0.001	−0.001	−0.001
	WH_1	WC	WO (1/2-all windows)	0.003	0.006	0.038
	WH_2	WO (1/2-all windows)	WC (back windows)	0.0003	0.001	−0.0003

^a^ WC (window closed); ^b^ WO (window opened); ^c^ AO (air conditioner is turned on).

**Table 5:** Explained variability (%) of influential factors in PM concentrations and their effects.

Travel Mode	Trip Name	Influential Factors (Statistical Significance)	Explained Variability			Significant Effects (Indicated by Factorial Analysis)
			PM_1_	PM_2.5_	PM_10_	
Bus	GZ (Evening-IT)	WC*PD ^a^	56.3% *	65.5% **	73.6% **	(1) When WC = 0, PD shows a significant effect: PM concentrations of group (PD = 4) are significantly higher than the PM concentrations of group (PD = 2) and group (PD = 5) (2) When PD = 2 and 4, WC shows a significant effect: when PD = 2, the PM_10_ concentration of group (WC = 0) is significantly higher than the PM_10_ concentration of group (WC = 1); when PD = 4, the PM_10_ concentration of group (WC = 0) is significantly lower than the PM_10_ concentrations of group (WC = 1)
		TD	13.4% *	14.4% *	16.8% **	Travel duration of each route segment is negatively correlated with the variation in PM concentrations
	SZ (Evening-OT)	Sit*PD ^a^	69.50% **	67.80% **	57.60% **	(1) When PD = 3 and PD = 4, Sit shows a significant effect: PM concentration of group (Sit = 1) is significantly higher than the PM concentration of group (Sit = 0) (2) When Sit = 0 or Sit = 1, PD shows significant effect: when Sit = 0, the PM concentration of group (PD = 3) is significantly higher than the PM concentration of group (PD = 4); when Sit = 1, the PM concentration of group (PD = 3) is significantly lower than the PM concentrations of group (PD = 4)
		IVL_RD	4.80% *	5.90% *	10.70% **	IVL_RD shows a negative effect: PM concentration is significantly higher when IVL_RD = 1 (near rear doors)
	SZ (Evening-IT)	PD*WC ^a^	60.8% **	61.7% **	60.0% **	PD shows a significant positive effect when WC = 1
		TD	9.8%*	9.7%*	11.2% *	Negative effect
	SZ (Morning-OT)	Sit*Vent_Near ^a^	/	/	52% **	When Sit = 1, PM concentration is significantly higher when Vent_Near = 1 compared to that when Vent_Near = 0
	WH (Morning-OT)	Sit*PD ^a^	54.60% *	/	/	(1) PD shows a significant positive effect when Sit = 1 (2) Sit does not show a significant interactive effect within each level of combination of PD effects shown.
Metro	CS (Morning-IT)	PD	46.40% **	54.10% **	/	Positive effect
	GZ (Morning-IT)	PD*IVL_Doors ^a^	66.60% **	62.00% *	/	Simple effects: (1) When PD = 5, PM concentration measured in the seating area is significantly higher than that measured near doors (2) In the seating area, PD shows a slight positive association with PM concentrations, but the relationship is not statistically significant.
	SZ (Evening-OT)	Vent_Near	58.40% **	57.50% **	/	PM concentration is significantly higher when Vent_Near = 1 compared to that when Vent_Near = 0
	WH (Morning-OT)	Sit	43.70% **	52.60% **	51.40% **	PM concentration is significantly higher when Sit = 1 compared to that when Sit = 0

^a^ regarding the interaction of factors, simple effect analysis was performed to test the simple effects of one factor within each level combination of the other effects shown. * Statistically significant (*p* < 0.05). ** Statistically significant (*p* < 0.01).

**Table 6:** Correlation coefficients of in-cabin particulate matter concentrations measured during the moving period and out-cabin and urban background environmental conditions compared by city.

			In-Cabin PM_1_	In-Cabin PM_2.5_	In-Cabin PM_10_
**Urban PM concentrations** **(Official)**	PM_10_	Bus	0.919 *	0.943 *	0.945 *
	PM_10_	Metro	0.914 *	0.916 *	-
**Out-Cabin PM concentrations** **(Self-measured)**	PM_1_	Bus (Platform)	0.982 **	0.986 **	-
	PM_2.5_		0.907 *	0.945 *	-
	PM_1_	Metro (Platform)	0.955 *	0.955 *	0.916 *
	PM_2.5_		0.941 *	0.944 *	0.909 *
	PM_10_		0.973 *	0.981 **	0.985 **
	PM_1_	Metro (T-P)	-	-	-
	PM_2.5_		-	-	-
	PM_10_		0.915 *	0.931 *	0.976 *
	PM_1_	Metro (E-T)	0.955 *	0.961 *	0.945 *
	PM_2.5_		0.955 *	0.963 *	0.949 *
	PM_10_		0.914 *	0.912 *	0.928 *
**Urban Meteorology** **(Official)**	PRE ^a^	Taxi	−0.986 **	−0.968 *	−0.922 *
	WS ^b^		−0.974 *	−0.994 **	−0.964 *

** Statistical significance is indicated at the 0.01 level (1-tailed). * Statistical significance is indicated at the 0.05 level (1-tailed). ^a^ PRE (precipitation between 8 and 20); and ^b^ WS (wind speed).

## Data Availability

Not applicable.

## Supplementary Materials

The following supporting information can be downloaded at: https://www.mdpi.com/article/10.3390/ijerph19105830/s1, Table S1: Field descriptions of the mobile application (survey app), Table S2: Description of data collected in this study, Table S3: Descriptive statistics on measurements of each transit in four cities, Table S4: Bus and metro trips selected for factorial analysis, Table S5: Results of factorial analyses for selected bus and metro trips. (a) Tests of Between-Subjects Effects. (b) Tests of Simple effects—Univariate Tests. (c) Tests of Simple effects—Pairwise Comparison.
