# Supplementary material for: A Comparison of Particulate Exposure Levels during Taxi, Bus, and Metro Commuting among Four Chinese Megacities
Source: Int J Environ Res Public Health. 2022 May 10;19(10):5830. doi: 10.3390/ijerph19105830 (PMC9140565; doi:10.3390/ijerph19105830)
Supplement: Supplementary file 1 [file ijerph-19-05830-s001.zip › ijerph-1650148-supplementary.pdf]

International Journal of
*Environmental Research and Public Health*

MDPI

**Table S1.** Field descriptions of the mobile application (survey app).

| In/outside-cabin | Field Name | Description |
|---|---|---|
| Outside-cabin (Bus & Metro) | Route Name (RN) | Name of bus/metro route |
| | Station Name (SN) | Name of metro station/bus stop where passengers were getting on/off |
| | Direction (DIR) | Direction of bus/metro travelling |
| | Level | Level of monitoring location at bus platform/metro station |
| | Location | (1) Bus: monitoring location at bus platform, i.e., left, middle, or right; (2) Metro: monitoring location in metro station, i.e., entrance/exit, turnstile, or platform |
| | Number of buses (NB) | Number of buses at the platform |
| | License Plate (LP) | License plate of the monitoring bus |
| | Coach number (CN) | Number of the coach (metro) that investigator gets on |
| | GPS positioning (GPS) | GPS positioning of investigators |
| In-cabin (Bus & Metro) | Station Name (SN) | Name of bus stop/metro station of each route segment while monitoring (bus/metro going through) |
| | Location in cabin | (1) Bus: Monitoring location inside the cabin, i.e., front, middle, rear door, or back region; (2) Metro: Monitoring location inside the cabin, i.e., near the doors, or seating area |
| | Vent | Monitoring location close to or far from vents |
| | Wind | Body feeling of wind, i.e., strong, weak, or no wind |
| | Window | Window nearing monitoring location is closed or open |
| | Location on route segment (LRS) | Location of bus/metro on each route segment while travelling, i.e., 1, 2, 3. |
| | Passenger density (PD) | Density of passengers at monitoring location |
| | Position | Investigator is sitting or standing while surveying |
| | GPS positioning | GPS positioning of investigators |
| In-cabin (Taxi) | Direction (DIR) | Direction of taxi travelling (inbound, outbound) |
| | Congestion | Yes or No |
| | Ventilation condition | Recorded by investigator, i.e. window condition (close or open), air conditioner (on or off) |

**Table S2.** Description of data collected in this study.

| Data Name | | City | Data records | Data attributes | Data source (format) |
|---|---|---|---|---|---|
| **PM concentration* (inside the cabin)** | **Taxi** | **SZ** | 9,070 | Time (hh:mm:ss), $PM_1$, $PM_{2.5}$, $PM_{10}$ concentrations. | Self-measured (.txt file) |
| | | **GZ** | 8,050 | | |
| | | **CS** | 16,928 | | |
| | | **WH** | 12,016(11,999) | | |
| | **Bus** | **SZ** | 10,499 (10,487) | | |
| | | **GZ** | 13,169(13,165) | | |
| | | **CS** | 13,608(13,559) | | |
| | | **WH** | 15,402(15,394) | | |
| | **Metro** | **SZ** | 5,695 | | |
| | | **GZ** | 6,837(6,830) | | |
| | | **CS** | 10,664 | | |
| | | **WH** | 10,661 | | |
| **Travel survey app** | **Bus** | **SZ** | 234 | Time (hh:mm:ss), App fields (Table S1), GPS positioning. | |
| | | **GZ** | 231 | | |
| | | **CS** | 293 | | |
| | | **WH** | 240 | | |
| | **Metro** | **SZ** | 158 | | |
| | | **GZ** | 169 | | |

| | | | | | |
|---|---|---|---|---|---|
| | | **CS** | 250 | | |
| | | **WH** | 228 | | |
| **In-transit Tem&RH** | **Taxi** | **SZ** | 155 | Time (hh:mm), temperature, and relative humidity. | |
| | | **GZ** | 139 | | |
| | | **CS** | 286 | | |
| | | **WH** | 205 | | |
| | **Bus** | **SZ** | 220 | | |
| | | **GZ** | 248 | | |
| | | **CS** | 279 | | |
| | | **WH** | 294 | | |
| | **Metro** | **SZ** | 201 | | |
| | | **GZ** | 208 | | |
| | | **CS** | 254 | | |
| | | **WH** | 293 | | |
| **Urban PM concentrations** | **Taxi** | **SZ** | 6,769 | Time (hh:mm:ss), $PM_1$, $PM_{2.5}$ and $PM_{10}$ concentrations (measured by investigators while walking to a bus platform or metro station and transferring between inbound and outbound trips.) | Self-measured (.txt file) |
| | | **GZ** | 3,041 | | |
| | | **CS** | 5,273 | | |
| | | **WH** | 7,157 | | |
| | **Bus** | **SZ** | 1,435 | | |
| | | **GZ** | 969 | | |
| | | **CS** | 1,724 | | |
| | | **WH** | 602 | | |
| | **Metro** | **SZ** | 2,664 | | |
| | | **GZ** | 2,310 | | |
| | | **CS** | 1,797 | | |
| | | **WH** | 1,460 | | |
| **Urban PM concentrations** | | **SZ** | 174 | Time (hh), $PM_{2.5}$ and $PM_{10}$ concentrations in morning and evening peak hours | Official urban fixed monitoring station (.txt) (https://air.cnemc.cn:18014/) |
| | | **GZ** | 156 | | |
| | | **CS** | 97 | | |
| | | **WH** | 124 | | |
| **Urban meteorological conditions** | | **SZ** | 9 | Day, temperature, relative humidity, precipitation, pressure, and wind speed. | RESDC(.txt) (https://www.resdc.cn/) |
| | | **GZ** | 9 | | |
| | | **CS** | 3 | | |
| | | **WH** | 9 | | |

* The data records of PM concentrations in brackets represent the number of records after data filtering (removing concentrations lower than 0).

**Table S3.** Descriptive statistics on measurements of each transit in four cities.

| | | | Taxi | | Bus | | Metro | |
|---|---|---|---|---|---|---|---|---|
| | | | Inbound | Outbound | Inbound | Outbound | Inbound | Outbound |
| SZ | # trips | | 2 | 2 | 2 | 2 | 2 | 2 |
| | TD (mins) | M | 35.31 | 34.00 | 36.60 | 45.23 | 22.57 | 24.42 |
| | | E | 37.85 | 43.93 | 41.03 | 52.05 | 23.42 | 24.45 |
| | $PM_1$ ($mg/m^3$) | M | 0.042 | 0.021 | 0.036 | 0.035 | 0.019 | 0.017 |
| | | E | 0.081 | 0.062 | 0.093 | 0.042 | 0.025 | 0.018 |
| | $PM_{2.5}$ ($mg/m^3$) | M | 0.045 | 0.023 | 0.038 | 0.037 | 0.021 | 0.019 |
| | | E | 0.087 | 0.068 | 0.097 | 0.044 | 0.027 | 0.019 |
| | $PM_{10}$ ($mg/m^3$) | M | 0.057 | 0.028 | 0.046 | 0.045 | 0.029 | 0.029 |
| | | E | 0.108 | 0.089 | 0.106 | 0.049 | 0.034 | 0.029 |
| | Tem (°C) | M | 26.55 | 26.75 | 25.39 | 24.77 | 23.69 | 27.17 |
| | | E | 26.20 | 26.96 | 25.54 | 24.88 | 23.43 | 26.98 |
| | RH | M | 74.54 | 73.90 | 74.74 | 72.26 | 74.66 | 64.02 |
| | | E | 72.29 | 69.75 | 74.28 | 70.73 | 67.47 | 62.36 |
| GZ | # trips | | 2 | 2 | 2 | 2 | 2 | 2 |
| | TD (mins) | M | 41.53 | 31.65 | 41.63 | 58.78 | 28.80 | 28.58 |
| | | E | 28.13 | 32.78 | 46.72 | 72.28 | 27.97 | 28.53 |

| | | | | | | | | |
|---|---|---|---|---|---|---|---|---|
| | $PM_1$ (mg/m$^3$) | M | 0.038 | 0.038 | 0.035 | 0.028 | 0.025 | 0.025 |
| | | E | 0.058 | 0.055 | 0.054 | 0.048 | 0.030 | 0.031 |
| | $PM_{2.5}$ (mg/m$^3$) | M | 0.043 | 0.043 | 0.037 | 0.030 | 0.027 | 0.028 |
| | | E | 0.064 | 0.061 | 0.059 | 0.050 | 0.032 | 0.033 |
| | $PM_{10}$ (mg/m$^3$) | M | 0.080 | 0.078 | 0.044 | 0.044 | 0.049 | 0.054 |
| | | E | 0.099 | 0.090 | 0.085 | 0.058 | 0.041 | 0.043 |
| | Tem (°C) | M | 22.76 | 23.86 | 25.36 | 24.62 | 27.02 | 28.04 |
| | | E | 26.04 | 26.53 | 29.32 | 25.68 | 25.31 | 25.93 |
| | RH | M | 70.25 | 68.58 | 66.18 | 64.71 | 62.54 | 58.15 |
| | | E | 63.20 | 61.60 | 57.06 | 60.21 | 60.71 | 59.06 |
| CS | # trips | | 2 | 2 | 2 | 2 | 2 | 2 |
| | TD (mins) | M | 81.45 | 55.47 | 48.93 | 68.32 | 45.95 | 43.47 |
| | | E | 91.77 | 53.38 | 58.30 | 51.18 | 45.03 | 43.22 |
| | $PM_1$ (mg/m$^3$) | M | 0.015 | 0.012 | 0.033 | 0.035 | 0.025 | 0.020 |
| | | E | 0.021 | 0.031 | 0.036 | 0.045 | 0.032 | 0.029 |
| | $PM_{2.5}$ (mg/m$^3$) | M | 0.015 | 0.012 | 0.035 | 0.038 | 0.026 | 0.020 |
| | | E | 0.022 | 0.034 | 0.039 | 0.050 | 0.033 | 0.030 |
| | $PM_{10}$ (mg/m$^3$) | M | 0.017 | 0.014 | 0.039 | 0.043 | 0.032 | 0.030 |
| | | E | 0.024 | 0.039 | 0.048 | 0.060 | 0.041 | 0.043 |
| | Tem (°C) | M | 23.30 | 15.01 | 15.50 | 13.11 | 16.80 | 18.14 |
| | | E | 19.06 | 22.05 | 19.24 | 15.69 | 15.52 | 19.66 |
| | RH | M | 64.92 | 82.38 | 57.36 | 76.94 | 63.45 | 62.07 |
| | | E | 57.85 | 47.71 | 66.77 | 61.56 | 56.13 | 52.85 |
| WH | # trips | | 2 | 2 | 2 | 2 | 2 | 2 |
| | TD (mins) | M | 45.07 | 43.65 | 53.42 | 67.30 | 43.75 | 44.22 |
| | | E | 67.58 | 43.90 | 79.65 | 60.40 | 45.12 | 44.53 |
| | $PM_1$ (mg/m$^3$) | M | 0.045 | 0.085 | 0.098 | 0.094 | 0.081 | 0.078 |
| | | E | 0.020 | 0.064 | 0.067 | 0.076 | 0.036 | 0.052 |
| | $PM_{2.5}$ (mg/m$^3$) | M | 0.055 | 0.099 | 0.120 | 0.110 | 0.089 | 0.086 |
| | | E | 0.024 | 0.078 | 0.086 | 0.108 | 0.039 | 0.057 |
| | $PM_{10}$ (mg/m$^3$) | M | 0.090 | 0.140 | 0.251 | 0.179 | 0.136 | 0.131 |
| | | E | 0.041 | 0.141 | 0.224 | 0.288 | 0.054 | 0.090 |
| | Tem (°C) | M | 22.52 | 18.50 | 18.26 | 19.79 | 19.44 | 18.20 |
| | | E | 19.97 | 19.83 | 22.31 | 18.73 | 21.08 | 19.85 |
| | RH | M | 56.83 | 57.00 | 55.00 | 52.26 | 58.41 | 56.27 |
| | | E | 51.33 | 54.54 | 52.90 | 51.65 | 57.30 | 57.30 |

**Table S4.** Bus and metro trips selected for factorial analysis.

| Travel mode | Trip information |
|---|---|
| Bus | 1) GZ— Three trips selected: inbound trip (IT) and outbound trip (OT) measured in evening peak, and outbound trip (OT) measured in morning peak, with normal distributions of $PM_1$ concentration, $PM_{2.5}$ concentration, and $PM_{10}$ concentration;<br>2) SZ—Three trips selected: inbound trip (IT) and outbound trip (OT) measured in evening peak, which shows a normal distribution of $PM_1$ concentration, $PM_{2.5}$ concentration, and $PM_{10}$ concentration, and outbound trip (OT) measured in morning peak, with normal distributions of $PM_{10}$ concentration;<br>3) WH—One trip selected: outbound trip (OT) measured in morning peak, which shows a normal distribution of $PM_1$ concentration. |
| Metro | 1) CS— 1 trip selected: inbound trip (IT) measured in morning peak, with normal distributions of $PM_1$ concentration and $PM_{2.5}$ concentration;<br>1) GZ— 1 trip selected: inbound trip (IT) measured in morning peak, with normal distributions of $PM_1$ concentration and $PM_{2.5}$ concentration;<br>2) SZ—1 trip selected: outbound trip (OT) measured in morning peak, which shows a normal distribution of $PM_{10}$ concentration; |

| | |
|---|---|
| | 3) WH—1 trip selected: outbound trip (OT) measured in morning peak, with normal distributions of $PM_1$ concentration, $PM_{2.5}$ concentration, and $PM_{10}$ concentration. |

**Table S5.** Results of factorial analyses for selected bus and metro trips.

(a) Tests of Between-Subjects Effects.

| Trip Name | Factors | Dependent variable | Type III Sum of Squares | F | Sig. |
|---|---|---|---|---|---|
| GZ-E-G (Bus) | WC*PD [a] | $PM_1$ | 0.00032 | 3.703 | 0.037 |
| | | $PM_{2.5}$ | 0.00067 | 6.526 | 0.006 |
| | | $PM_{10}$ | 0.00564 | 15.375 | 0.000 |
| | Corrected Total | $PM_1$ | 0.00056 | | |
| | | $PM_{2.5}$ | 0.00102 | | |
| | | $PM_{10}$ | 0.00766 | | |
| SZ-E-B (Bus) | Sit*PD [a] | $PM_1$ | 0.00178 | 19.827 | 0.000 |
| | | $PM_{2.5}$ | 0.00187 | 19.866 | 0.000 |
| | | $PM_{10}$ | 0.00208 | 15.301 | 0.000 |
| | IVL_RD | $PM_1$ | 0.00012 | 5.527 | 0.038 |
| | | $PM_{2.5}$ | 0.00016 | 6.928 | 0.023 |
| | | $PM_{10}$ | 0.00039 | 11.405 | 0.006 |
| | Corrected Total | $PM_1$ | 0.00256 | | |
| | | $PM_{2.5}$ | 0.00276 | | |
| | | $PM_{10}$ | 0.00362 | | |
| SZ-E-G (Bus) | PD*WC [a] | $PM_1$ | 0.00540 | 11.091 | 0.001 |
| | | $PM_{2.5}$ | 0.00607 | 11.889 | 0.001 |
| | | $PM_{10}$ | 0.00742 | 11.172 | 0.001 |
| | Corrected Total | $PM_1$ | 0.00889 | | |
| | | $PM_{2.5}$ | 0.00984 | | |
| | | $PM_{10}$ | 0.01237 | | |
| SZ-M-B (Bus) | Sit*Vent_Near [a] | $PM_{10}$ | 0.00052 | 7.577 | 0.006 |
| | Corrected Total | $PM_{10}$ | 0.00099 | | |
| WH-M-B (Bus) | Sit*PD [a] | $PM_1$ | 0.00067 | 4.211 | 0.019 |
| | Corrected Total | $PM_1$ | 0.00123 | | |
| CS-M-G (Metro) | PD | $PM_1$ | 0.000019 | 4.332 | 0.022 |
| | | $PM_{2.5}$ | 0.000022 | 5.890 | 0.007 |
| | Corrected Total | $PM_1$ | 0.000042 | | |
| | | $PM_{2.5}$ | 0.000041 | | |
| GZ-M-G (Metro) | PD*IVL_Doors [a] | $PM_1$ | 0.000052 | 8.974 | 0.007 |
| | | $PM_{2.5}$ | 0.000053 | 7.336 | 0.013 |
| | Corrected Total | $PM_1$ | 0.000079 | | |
| | | $PM_{2.5}$ | 0.000085 | | |
| SZ-E-B (Metro) | Vent_Near | $PM_1$ | 0.000210 | 12.614 | 0.006 |
| | | $PM_{2.5}$ | 0.000215 | 12.158 | 0.007 |
| | Corrected Total | $PM_1$ | 0.000359 | | |
| | | $PM_{2.5}$ | 0.000374 | | |
| WH-M-B (Metro) | Sit | $PM_1$ | 0.000304 | 11.596 | 0.004 |
| | | $PM_{2.5}$ | 0.000403 | 16.675 | 0.001 |
| | | $PM_{10}$ | 0.00096 | 15.879 | 0.001 |
| | Corrected Total | $PM_1$ | 0.000696 | | |
| | | $PM_{2.5}$ | 0.000766 | | |
| | | $PM_{10}$ | 0.001866 | | |

[a] regarding the interaction of factors, simple effect analysis was performed to test the simple effects of one factor within each level combination of the other effects shown. Tests of Simple effects are shown in tables (b) and (c).

(b) Tests of Simple effects - Univariate Tests

| Trip Name | Dependent Variable | Factor | | Sum of Squares | F | Sig |
|---|---|---|---|---|---|---|
| GZ-E-G (Bus) | $PM_1$ | WC=0 | Contrast | .000 | 6.589 | .015 |
| | | | Error | .000 | | |
| | | PD=4 | Contrast | .000 | 8.360 | .016 |
| | | | Error | .000 | | |
| | $PM_{2.5}$ | WC=0 | Contrast | .001 | 12.632 | .002 |
| | | | Error | .000 | | |
| | | PD=4 | Contrast | .000 | 10.896 | .008 |
| | | | Error | .000 | | |
| | $PM_{10}$ | WC=0 | Contrast | .005 | 33.090 | .000 |
| | | | Error | .001 | | |
| | | PD=2 | Contrast | .001 | 7.169 | .023 |
| | | | Error | .001 | | |
| | | PD=4 | Contrast | .001 | 8.871 | .014 |
| | | | Error | .001 | | |
| SZ-E-B (Bus) | $PM_1$ | PD=3 | Contrast | .000 | 6.186 | .000 |
| | | | Error | .000 | | |
| | | PD=4 | Contrast | .002 | 73.928 | .000 |
| | | | Error | .000 | | |
| | | Sit=0 | Contrast | .000 | 18.072 | .001 |
| | | | Error | .000 | | |
| | | Sit=1 | Contrast | .000 | 5.219 | .025 |
| | | | Error | .000 | | |
| | $PM_{2.5}$ | PD=3 | Contrast | .000 | 6.036 | .032 |
| | | | Error | .000 | | |
| | | PD=4 | Contrast | .002 | 74.014 | .000 |
| | | | Error | .000 | | |
| | | Sit=0 | Contrast | .000 | 17.849 | .001 |
| | | | Error | .000 | | |
| | | Sit=1 | Contrast | .000 | 5.485 | .022 |
| | | | Error | .000 | | |
| | $PM_{10}$ | PD=3 | Contrast | .000 | 5.948 | .033 |
| | | | Error | .000 | | |
| | | PD=4 | Contrast | .002 | 55.424 | .000 |
| | | | Error | .000 | | |
| | | Sit=0 | Contrast | .000 | 11.498 | .006 |
| | | | Error | .000 | | |
| | | Sit=1 | Contrast | .000 | 4.568 | .036 |
| | | | Error | .000 | | |
| SZ-E-G (Bus) | $PM_1$ | WC=1 | Contrast | .005 | 15.393 | .000 |
| | | | Error | .002 | | |
| | $PM_{2.5}$ | WC=1 | Contrast | .006 | 16.175 | .000 |
| | | | Error | .002 | | |
| | $PM_{10}$ | WC=1 | Contrast | .007 | 15.025 | .001 |
| | | | Error | .003 | | |
| SZ-M-B (Bus) | $PM_{10}$ | Sit=1 | Contrast | .000 | 8.403 | .012 |
| | | | Error | .000 | | |
| WH-M-B (Bus) | $PM_1$ | Sit=1 | Contrast | .001 | 7.965 | .005 |
| | | | Error | .001 | | |
| GZ-M-G (Metro) | $PM_1$ | PD=5 | Contrast | .000 | 14.236 | .004 |
| | | | Error | .000 | | |
| | $PM_{2.5}$ | PD=5 | Contrast | .000 | 12.056 | .007 |
| | | | Error | .000 | | |

(c) Tests of Simple effects - Pairwise Comparison

| Trip Name | Dependent Variable | Factor | | | Mean Difference (I-J) | Sig |
|---|---|---|---|---|---|---|
| GZ-E-G (Bus) | $PM_1$ | WC=0 | (I)PD=2 | (J)PD=4 | .012 | .025 |
| | | | (I)PD=4 | (J)PD=5 | -.010 | .038 |
| | | PD=4 | (I)WC=0 | (J)WC=1 | -.016 | .016 |
| | $PM_{2.5}$ | WC=0 | (I)PD=2 | (J)PD=4 | .021 | .002 |
| | | | (I)PD=4 | (J)PD=5 | -.012 | .029 |
| | | PD=4 | (I)WC=0 | (J)WC=1 | -.020 | .008 |
| | $PM_{10}$ | WC=0 | (I)PD=2 | (J)PD=4 | .063 | .000 |
| | | | (I)PD=2 | (J)PD=5 | .046 | .000 |
| | | PD=2 | (I)WC=0 | (J)WC=1 | .023 | .023 |
| | | PD=4 | (I)WC=0 | (J)WC=1 | -.034 | .014 |
| SZ-E-B (Bus) | $PM_1$ | Sit=0 | (I)PD=3 | (J)PD=4 | .017 | .004 |
| | | PD=3 | (I)Sit=0 | (J)Sit=1 | -.009 | .030 |
| | | PD=4 | (I)Sit=0 | (J)Sit=1 | -.035 | .000 |
| | $PM_{2.5}$ | Sit=0 | (I)PD=3 | (J)PD=4 | .018 | .001 |
| | | Sit=1 | (I)PD=3 | (J)PD=4 | -.009 | .046 |
| | | PD=3 | (I)Sit=0 | (J)Sit=1 | -.009 | .032 |
| | | PD=4 | (I)Sit=0 | (J)Sit=1 | -.035 | .000 |
| | $PM_{10}$ | Sit=0 | (I)PD=3 | (J)PD=4 | .017 | .006 |
| | | PD=3 | (I)Sit=0 | (J)Sit=1 | -.010 | .033 |
| | | PD=4 | (I)Sit=0 | (J)Sit=1 | -.037 | .000 |
| SZ-E-G (Bus) | $PM_1$ | WC=1 | (I)PD=2 | (J)PD=3 | -.026 | .037 |
| | | | (I)PD=3 | (J)PD=4 | .041 | .000 |
| | $PM_{2.5}$ | WC=1 | (I)PD=2 | (J)PD=3 | -.028 | .030 |
| | | | (I)PD=3 | (J)PD=4 | .042 | .000 |
| | $PM_{10}$ | WC=1 | (I)PD=2 | (J)PD=3 | -.034 | .021 |
| | | | (I)PD=3 | (J)PD=4 | .046 | .001 |
| SZ-M-B (Bus) | $PM_{10}$ | Sit=1 | (I)Vent_Near=0 | (J)Vent_Near=1 | -.011 | .012 |
| WH-M-B (Bus) | $PM_1$ | Sit=1 | (I)PD=1 | (J)PD=3 | .023 | .015 |
| | | | (I)PD=2 | (J)PD=3 | .013 | .035 |
| GZ-M-G (Metro) | $PM_1$ | PD=5 | (I)IVL_Doors=0 | (J)IVL_Doors =1 | .005 | .004 |
| | $PM_{2.5}$ | PD=5 | (I)IVL_Doors=0 | (J)IVL_Doors =1 | .006 | .007 |
